# The Empowerment of Adolescents with Type 1 Diabetes Is Associated with Their Executive Functions

**DOI:** 10.1155/2019/5184682

**Published:** 2019-04-30

**Authors:** Włodzimierz Łuczyński, Izabela Łazarczyk, Ilona Szlachcikowska, Żaneta Kiernozek, Anna Kaczmarek, Oliwia Szylaj, Agnieszka Szadkowska, Przemysława Jarosz-Chobot, Barbara Głowińska-Olszewska, Artur Bossowski

**Affiliations:** ^1^Department of Pediatrics, Endocrinology, Diabetology with Cardiology Division, Medical University of Białystok, Białystok 15-274 Waszyngtona 24, Poland; ^2^Students' Scientific Section of the Department of Pediatrics, Endocrinology, Diabetology with Cardiology Division, Medical University of Białystok, Białystok 15-274 Waszyngtona 24, Poland; ^3^Department of Pediatrics, Oncology, Hematology and Diabetology, Medical University of Łódź, 91-738 Łódź, Sporna 36/50, Poland; ^4^Department of Pediatric Diabetology, Silesian Medical University of Katowice, 40-752 Katowice, Medyków 16, Poland

## Abstract

**Background:**

Adolescence is a difficult period for young people with type 1 diabetes mellitus (T1DM), both in psychological and clinical terms. Empowerment therapy may support these patients, provided they are ready to change and have adequate executive functions to facilitate this change. Therefore, we hypothesise that the readiness of adolescents with T1DM to change is related to clinical features and/or their executive functions.

**Methods:**

Using the Diabetes Empowerment Scale and the Behavioural Rating Inventory of Executive Function, we evaluated patients with T1DM duration of more than one year from three Polish diabetes centres of the PolPeDiab study group (N = 146). We related the data to features associated with disease and treatment and compared the results to those of adolescents without diabetes (N = 110).

**Results:**

We observed that adolescents with T1DM had a higher rate of abnormal results in executive function tests than their peers without diabetes (p > 0.05). Diabetes empowerment in this group of patients decreased with disease duration (r = -0.25, p = 0.006) and increased with deteriorating metabolic control (HbA1c; r = 0.25, p = 0.006). The greater the deficiencies in executive functions among adolescents with T1DM, the greater their readiness to change. The relationship between executive functions and diabetes empowerment is partially gender-differentiated.

**Conclusions:**

To conclude, we propose individualized diabetes education in this group of patients based on the assessment of readiness to change and executive functions.

## 1. Introduction

The increased incidence of type 1 diabetes mellitus (T1DM) among children is an important public health burden [1, 2]. Adolescence is a difficult period for young patients with T1DM, both in psychological and clinical terms including self-management [3]. Unfortunately, adolescents with T1DM frequently fail to comply with the recommendations for managing their diabetes, which leads to inadequate metabolic control and earlier disease complications [4]. An additional challenge for adolescents managing their disease has been the introduction of new technologies, such as advanced insulin pumps, glycaemic sensors, bolus calculator functions, and a variety of software available to support the decision-making process. Therefore, to properly manage their disease, adolescents with T1DM should have good cognitive abilities. Major cognitive processes include planning, organizing, initiation, shifting, cognitive set, memory, monitoring, and emotional control [5], which are collectively termed executive functions.

In recent years, empowerment has been proposed as a therapeutic process, aimed to increase the patient's own ability to think critically and act autonomously [6]. This type of therapy can be effective in both diabetes and obesity [7], although previous studies on empowerment therapy have primarily involved adult patients with type 2 diabetes (T2DM) who take oral medications. Despite its potential benefit, empowerment therapy among T1DM adolescents with inadequate metabolic control is difficult to implement and requires prior evaluation of the patients' readiness to change in terms of self-management, diet, and insulin dosage [8]. Moreover, the patients' readiness to change, and thus the effectiveness of empowerment therapy, may be influenced by their executive functions, something which has not been studied to date.

Our experience shows that adolescents with T1DM frequently want to change their lifestyle with respect to diabetes, but they often do not achieve their long-term goals. We suggest that deficits in their executive functions may be responsible for that. Therefore, we hypothesised that the readiness to change of adolescent patients with T1DM is related to their clinical features and executive functions. Using the Diabetes Empowerment Scale (DES) and the Behavioural Rating Inventory of Executive Function (BRIEF), we evaluated adolescent patients from three Polish diabetes centres. We related the data to features associated with the disease and its treatment and compared the results to those obtained for adolescents without diabetes. We believe that the results might help better understand the difficulties in diabetes education in young patients, and how this education should be adapted to match their capabilities and meet their needs for an improved quality of life.

## 2. Patients and Methods

A cross-sectional study was conducted between October 2015 and June 2018 in three Polish diabetes centres of the PolPeDiab group. The criteria for inclusion in the study group were age ≥14 years to ≤18 years, diagnosis of T1DM, end of remission, possible coexistence and treatment for immunological diseases of the thyroid gland and/or gastrointestinal tract and hypertension/albuminuria. The comparison group consisted of adolescent patients without diabetes, admitted to one of our pediatric departments for check-up tests due to cardiac problems (clinically irrelevant heart defects or arrhythmias). Adolescents from the comparison group did not take any medications. Psychiatric abnormalities and evidence of chromosomal disorders in physical examination were excluded in both the study and comparison groups.

The study design was approved by the Ethics Committee at the Medical University of Bialystok in accordance with the Declaration of Helsinki (No. R-I-002/374/2014). Signed informed consent was obtained from patients and their parents/guardians. The rates of consent were 93.0% and 86.1% in the study and comparison groups, respectively.

The following parameters were evaluated in all adolescents: age, sex, and body mass index standardized deviation score (BMI-SDS). Body mass index (BMI) was calculated from the height and weight measured by appropriately trained members of the research group. The BMI-SD referred to the centile charts for gender, age, and BMI [9]. In addition, the following disease-associated parameters were evaluated in the group with diabetes: disease duration, treatment regimen (pens versus pumps), mean daily insulin use (U/kg/day, mean from the last 3 days), glycosylated hemoglobin levels (DCCT Units, mean for the last year of treatment, minimum 4 measurements), and mean number of assessments of glucose with glucose meter/day. To calculate the daily number of insulin units per kg of body weight (IU/kg), the available medical documentation and pump software data were considered. Glycated hemoglobin (HbA1c) was measured in venous whole blood collected in EDTA. It was measured in a biochemical analyser, using an immunoassay with monoclonal antibodies. We also noted the presence of celiac disease/use of gluten-free diet, hypertension/nephropathy, and thyroid gland diseases, including treatment for these conditions. Hypothyroidism, celiac disease, hypertension, and nephropathy were reported based on the current criteria.

The empowerment of the participants was measured using the Diabetes Empowerment Scale [10], reflecting three domains: “managing the psychosocial aspects of diabetes” (DES I), “assessing dissatisfaction and readiness to change” (DES II), and “setting and achieving diabetes goals” (DES III) (grant number P30DK020572 MDRC from the National Institute of Diabetes and Digestive and Kidney Diseases). Higher mean scores in DES mean greater diabetes empowerment.

Cognitive functions were assessed in all adolescents with the Behavioural Rating Inventory of Executive Functions® – Self Report Version (BRIEF®-SR) scale. BRIEF®-SR is an 80-item standardized self-report measure developed to capture older children's and adolescents' views of their own executive functions, or self-regulation, in their everyday environment [11]. Lower scores indicate better executive functioning. Values ≥65 are considered abnormally elevated [11]. Only children with all data available were qualified for analysis.

Data are presented as means and standard deviation (SD) and rates of incidence of a given characteristic in the evaluated group. Univariate analysis was conducted using the* Mann*–*Whitney* U* test* for continuous variables and the Chi-square test for the nominal ones. Correlations were performed using Spearman's correlation. Multivariate adjusted linear and logistic regressions were used to evaluate the impact of clinical features and cognitive functions on Diabetes Empowerment Scale results. A p < 0.05 was considered statistically significant. Statistical analysis was performed using the Statistica 13 software (StatSoft, Tulsa, OK, USA).

## 3. Results

Anthropometric data of both groups and clinical data concerning the disease and treatment of adolescents with T1DM are presented in Table 1. Participants from the comparison group did not differ significantly from their peers with diabetes in terms of age, sex distribution, and standardized BMI (all p > 0.05).

### 3.1. Executive Functions

The rates of normal and deficient results in the executive function tests were compared between the groups. Significantly higher proportions of T1DM adolescents with impaired executive functions (score ≥65) were noted compared to those without diabetes in the “organization of materials” function (31 [21.0%] versus 9 [8.0%], respectively; p = 0.01) and in the Global Executive Composite (GEC), which summarizes all executive functions (42 [28.5%] versus 16 [14.2%], respectively; p = 0.01). No gender differences were found in these scores. The remaining rates of deficient results and mean values of executive functions in patients with T1DM did not differ significantly from those without T1DM (Table 2).

No correlation was observed between the executive functions among adolescents with diabetes and age, sex, disease duration, number of daily glycaemic measurements, glycaemic control, insulin delivery tool (pens versus pump), BMI, or comorbidities (p > 0.05 in all cases).

### 3.2. Readiness to Change and Executive Functions

The mean values obtained were 2.21±0.5 for DES I, 2.15±0.37 for DES II, and 2.21±0.45 for DES III. The mean total DES score was 2.19±0.37. DES scores in the group of adolescents with T1DM did not show correlations with: sex, age, standardized BMI, insulin regimen (pumps versus pens), or accompanying diseases. Diabetes empowerment was also not associated with the number of daily glucose assessments in the study group (p > 0.05).

The results for DES I (“managing the psychosocial aspects of diabetes”) were related to disease duration (r = -0.25, p = 0.006): the shorter the duration of the disease, the higher the diabetes empowerment. Diabetes duration was associated with the DES total score (r = -0.23, p = 0.01). In addition, DES I and DES total scores were correlated with the HbA1c value as follows: the poorer the metabolic control, the higher the empowerment (r = 0.25, p = 0.006). Furthermore, the DES total score was related to the daily insulin use calculated per day and per patient body weight, i.e., the lower the insulin dose, the higher the diabetes empowerment (DES score; r = -0.22, p = 0.01).

In the correlation analysis, numerous relationships were noted between the executive functions (BRIEF-SR scale) and the readiness to change as assessed in the DES scale (Table 3). These correlations were positive, which means that weaker executive function skills were associated with greater diabetes empowerment. However, the results of the DES II domain (“assessing dissatisfaction and readiness to change”) showed correlation only with the executive function “organization of materials.” In further analysis, considering the sex of adolescents with T1DM, this correlation was significant in boys (r = 0.33, p < 0.01), but not in girls (r = 0.1, p > 0.05). However, the remaining correlations of executive functions with the results of DES I and III and the total score were statistically significant in both boys and girls (p < 0.01). Similar relationships were noted in the analysis of the DES results with respect to normal and abnormal results of executive functions: patients with deficient results in all executive functions were characterized by significantly higher values in DES I, III, and total scores, but not in DES II. For example, the total DES score in the group with normal GEC was 2.08±0.3, whereas in the group with abnormal GEC the score was - 2.4±0.4 (p = 0.001).

Finally, using the regression model, we tested our primary hypothesis that executive functions and clinical factors (age, sex, metabolic control, self-management, treatment regimen, insulin dose) are related to the readiness to change in adolescents with diabetes. The variability of DES I, III, and DES total score was explained by all executive functions as well as disease duration, metabolic control, and insulin dose (R = 0.59, R^∧^2 = 0.35, p < 0.001). These associations were maintained when patients were grouped by sex.

In contrast, the variability of DES II (“assessing dissatisfaction and readiness to change”) was explained only by the executive function “organization of materials” and the dose of insulin/kg/day (R = 0.42, R^∧^2 = 0.18, p < 0.01). When the patients were grouped by sex, readiness to change in boys was solely associated with the “organization of materials” function, while in girls DES II was correlated only with the insulin dose per day.

## 4. Discussion

In our experience, reeducation (including that based on empowerment) produces desired results in some adolescents with diabetes, while in others its effect is only temporary or none. To determine ways to increase the effectiveness of educational programs in diabetes, we investigated the factors that influence the readiness to change among adolescents with T1DM. We observed a higher rate of abnormal executive functioning among adolescents with T1DM compared to their peers without diabetes. At the same time, diabetes empowerment was associated with executive functions, disease duration, metabolic control, and insulin doses. We also observed gender-related differences in some of these relationships.

While the influence of executive functioning on the adherence to medical recommendations or metabolic control has been previously evaluated (reviewed in [5]), its effects on the readiness to change among adolescents with T1DM have not been studied so far. One report showed that the relationship between adherence to a diabetes regimen and executive functioning among children with T1DM was not age-dependent [12]. This observation was surprising, as older teenagers were thought to be more independent from their parents and their care, and thus more responsible for their actions. Therefore, in theory, the executive functioning of teenagers should affect their metabolic control to a higher degree than in younger children. However, perhaps the decisive factor is not age itself, but the degree of responsibility that young people have for themselves and their health.

It is difficult to evaluate the effect of executive functions on diabetes care and thus a patient's ability to count calories and insulin doses and adjust insulin to effort, and so on. Indirectly, this effect is assessed by the relationship with metabolic control. The authors of one study have stated that larger deficits in the executive functions of adolescents with T1DM lead to worse adherence to recommendations and lower quality of life [13]. However, they failed to find a direct relationship between executive functioning and metabolic control, or the number of glycaemic measurements. Similarly, we did not confirm such a direct relationship in our study, which suggests that this relationship may be influenced by other factors. While the age of the patients in the previous study was similar to that of our group (13–17 years), metabolic control was poorer (mean HbA1c = 9.16), the number of glycaemic measurements was lower (3.29/day), and only half of the patients were treated with a personal insulin pump.

What is the probable cause of executive function deficit in patients with T1DM? Whether hyperglycaemia in the course of diabetes impairs executive functions and subsequently weakens adherence to therapy recommendations, or the initial impairment of executive functions leads to not following care guidelines and the deterioration of metabolic control, remains unclear [14]. McNally et al. proposed that better executive functioning leads to better adherence to therapy recommendations, which results in more adequate metabolic control [15]. However, we did not observe this relationship. Instead, we found that poorer results in executive functioning correlated with higher diabetes empowerment. Moreover, in a 2-year prospective study, the results in executive functions did not change and did not predict changes in self-management and metabolic control in children with T1DM aged 9–11 years [16]. Nevertheless, the results of the abovementioned studies are not mutually exclusive. One possible explanation for our results may be that adolescents with impaired executive functioning are somehow aware of their deficiencies and, therefore, readier to change.

The association between executive functioning and empowerment can be mediated by adherence. One study showed better metabolic control in patients with better executive functioning, but only in adolescents reporting good adherence [17]. In adolescents reporting poor adherence, the correlation was reversed: better metabolic control was observed in adolescents with more abnormal executive functioning. In addition, the authors observed a phenomenon known from everyday diabetes practice: in those with lower adherence, parents believed that children were responsible for their own diabetes therapy, while the children expected their parents to be fully responsible. Certainly, more attention should be paid to communicating with young people with T1DM to make them aware of the benefits of diabetes education, as some of them are not sufficiently involved in this process [18].

We also observed interesting gender-related differences in the relationships between executive functions and diabetes empowerment. “Assessing dissatisfaction and readiness to change” was related to the executive function “organization of materials” exclusively in boys, with poorer executive functioning correlating with higher diabetes empowerment. Similarly, a previous study showed that poorer executive function in boys correlated with better self-reported adherence [13]. Conversely, in girls, larger problems in executive function resulted in worse self-reported adherence. Indeed, the results of the BENCH-D study indicate that diabetes-related stress correlates with metabolic control in women but not in men [19]. In another regression analysis, which included the influence of disease duration, higher emotion regulation difficulties were associated with higher HbA1c in boys but not girls [20]. Another study showed that deficits in executive functions in adolescents with T1DM were associated with inadequate metabolic control, increased number of visits to the clinic, and lower physical activity [21]. Interestingly, self-reported problems with executive functions were common among girls, but those reported by parents were more common in boys. Similarly, in a Swedish study using DES, women with T2DM reported higher education support needs than men [22]. Despite these differences, research on a larger number of patients is needed to explain the effect of gender on the relationship between readiness to change and the executive functions of adolescents with T1DM. Nonetheless, there is certainly a relationship between gender and the parameters examined, and this should be considered when educating adolescents with diabetes.

The results of studies on the readiness to change and its relationship with age, duration of the disease, and metabolic control are diverse. In a study conducted on adult patients with T2DM, “assessing dissatisfaction and readiness to change” and “setting and achieving diabetes goals” decreased with the patient's age; the first parameter also decreased with the disease duration [23]. In a regression analysis involving a similar group of patients, diabetes empowerment was positively influenced by education and good metabolic control [24]. In an educational program based on empowerment, a statistically significant improvement in metabolic control was observed only among adolescents who participated in the program together with their parents (HbA1c: 8.9% versus 7.6%; p < 0.05); however, the size of this group was small [25]. The authors of this report concluded that parental participation is necessary to obtain therapeutic effects, with which we agree. This conclusion seems to concern not only empowerment, but also all types of education in adolescents with diabetes. These reports highlight the main problems of empowerment therapy among adolescents with T1DM, including a lack of willingness to participate in the program, a small number of patients involved, a small number of parents taking active part in the therapy, a high rate of inadequate metabolic control in this group of patients, variability of HbA1c over time, and unexplained temporary deterioration or improvement of metabolic control.

Some data in this field refer to type 2 diabetes. In a large group of Chinese patients with T2DM, empowerment was predictive of metabolic control and self-management of patients in a manner that was independent of age, sex, marital status, education level, and disease duration [26]. In other studies, the results of DES correlated with age, education level, disease duration, the diabetes education program applied, and metabolic control among adult patients with T2DM [27, 28]. In the BENCH-D study previously cited, people who obtained a result in the upper quartile (meaning “more ready to change”) in DES-SF were younger, more often male, with a higher level of education, with better metabolic control, and a lower rate of distant complications compared to patients from other quartiles [29]. It is possible that diabetes empowerment would increase after reeducation in all patients with diabetes, including adults and children with either T1DM or T2DM. Indeed, when the PRIMAS education program (which includes elements of empowerment) was used in adults with T1DM, improvements were observed in metabolic control parameters, as well as in DES and patient satisfaction with insulin treatment [30]. However, this study was not blind, and the observation time was quite short.

One of the advantages of our study is the large number of patients and participants in the comparison group. Other advantages include the evaluation of the relationship between readiness to change and executive functions, which was performed for the first time, and the building of a regression model to explain factors that influence the diabetes empowerment of adolescents with T1DM. Nonetheless, our research also has several limitations, which should be considered when interpreting the results. First, since this is a cross-sectional study, a causal relationship in the studied associations cannot be proved. Such a relationship can be proved only in a prospective study. It also seems that the assessment of executive functions should be performed at the time of the disease onset, as the effect of fluctuations in glucose levels (characteristic of T1DM) on the results of executive function tests is unknown. Furthermore, we used the Polish version of DES, which has not been validated. Another limitation is the lack of data from parents about the executive functions of their children (adolescents). It is also difficult to assess whether accompanying diseases affect DES or executive functions, because the group with comorbidities was small and disease stabilization was a qualification criterion. However, thanks to good diabetes care, only a few adolescents with T1DM had unstable accompanying diseases.

The management of diabetes is complicated. It requires accurate insulin dosing with personal insulin pumps, counting carbohydrate and protein-fat exchanges, obtaining multiple blood glucose measurements, and adjusting insulin doses to physical effort. Considering the complexity of this system, it is not surprising that some adolescents have difficulty complying with the rules, which leads to fluctuations in blood glucose and poor metabolic control. Whether empowerment therapy leads to a better quality of life and reduces the risk of diabetes-related complications in adolescents remains unclear. It could be that adolescents with executive function deficits require a simpler approach to diabetes therapy and greater parental support. Perhaps the use of a bolus calculator function could be a simple solution for patients treated with a personal insulin pump and that have impaired executive functions. In some rare cases (very poor metabolic control and/or self-control and/or cooperation with diabetes team, etc.) insulin pump therapy does not improve quality of life and metabolic control and should be replaced by pen therapy with fixed doses of insulin. In spite of that, the results of our study indicate that the poorer the executive function, the greater the diabetes empowerment. This surprising observation may indicate the need to evaluate the executive functions prior to implementing complicated diabetes therapies and to personalize diabetes education. All adolescents with diabetes should receive care adjusted to their needs and capabilities and obtain support from an experienced integrated therapy team that is sensitive to the specific nature of this chronic disease and the problems of adolescence. It seems that to improve the quality of life and metabolic control in this group of patients the latest technologies should be implemented, and more time devoted to adolescents with diabetes and their families. Therefore, implementing a guided self-determination approach (e.g., based on empowerment) that supports problem-solving and decision-making related to insulin therapy in adolescents with T1DM may improve their motivation to manage their diabetes care [8].

## 5. Conclusions

The results of our research indicate that adolescents with T1DM are characterized by a higher rate of abnormal results in executive function tests compared to their peers without diabetes. Diabetes empowerment in this group of patients depends on the duration of the disease, metabolic control, and executive functions, and the relationships are partially gender-differentiated. We recommend assessment of readiness to change and executive functions in order to individualize and adapt therapy to the needs and abilities of adolescents with T1DM. The impact of such measures on the quality of life and metabolic control in this group of patients requires further research.

## Figures and Tables

**Table 1:** Clinical features of the comparison group and adolescents with T1DM included in the study.

	Comparison group	Adolescents with T1DM
Number of patients	N=112	N=147

Sex Male / Female	52 (46.4%) / 60 (53.6%)	69 (47.0%) / 78 (53.0%)

Age in years (mean ±SD)	15.8 ± 1.6	16.0 ± 1.4

SDS-BMI (mean ±SD)	0.78 ± 1.3	0.73 ± 1.2

Age of diabetes onset (mean ±SD)	-	8.7 ± 3.7

Disease duration (mean ±SD)	-	7.2 ± 3.7

Therapy: pumps / pens*∗*	-	116 (78.9%) / 31 (21.1%)

HbA1c % (mean ±SD)	-	8.5 ± 2.3%

Dose of insulin (mean U/kg/day)	-	0.78

Glycaemia measurements / day (mean ±SD)	-	5.6 ± 2.2

Celiac disease = gluten free diet	-	14 (9.5%)

Hashimoto disease	-	23 (15.6%)

Hypertension / nephropathy	-	6 (4.0%)

There were no statistically significant differences between adolescents treated with the pens and with the pump in the clinical parameters mentioned in Table 1.

**Table 2:** Comparison of executive functions between adolescents with and without T1DM. All differences were not statistically significant (p > 0.05).

Scale / Index	Comparison group	Adolescents with type 1 diabetes
Inhibit	56.4 ± 10.1	58.0 ± 10.5

Shift	54.6 ± 10.5	54.5 ± 11.0

Emotional control	58.4 ± 10.9	60.5 ± 11.9

Monitor	53.3 ± 9.9	53.9 ± 10.4

Behaviour regulation index BRI	57.4 ± 10.5	58.9 ± 10.8

Working memory	54.5 ± 9.4	56.3 ± 10.8

Plan organize	51.9 ± 9.8	53.4 ± 11.7

Organisation of materials	50.7 ± 9.5	53.8 ± 11.2

Task completion	53.0 ± 10.0	55.1 ± 9.9

Metacognition index MI	53.1 ± 9.1	55.5 ± 10.7

GEC (BRI+MI)	55.7 ± 9.5	57.7 ± 10.8

Subscale		

Behavioural shift	55.9 ± 10.8	55.0 ± 11.3

Cognitive shift	52.3 ± 10.8	53.6 ± 11.5

**Table 3:** Correlations between executive functions and readiness to change in T1DM adolescents. DES I – managing the psychosocial aspects of diabetes, DES II – assessing dissatisfaction and readiness to change, DES III – setting and achieving diabetes goals.

Diabetes empowerment				
Executive function	DES I	DES II	DES III	DES total score
Inhibit	0.27*∗∗*	-0.04	0.18*∗*	0.22*∗*

Shift	0.36*∗∗*	0.06	0.33*∗∗*	0.36*∗∗*

Emotional control	0.30*∗∗*	-0.16	0.20*∗*	0.22*∗*

Monitor	0.30*∗∗*	0.18	0.28*∗∗*	0.33*∗∗*

Working memory	0.32*∗∗*	0.11	0.31*∗∗*	0.33*∗∗*

Plan organize	0.46*∗∗*	0.14	0.49*∗∗*	0.50*∗∗*

Organisation of materials	0.32*∗∗*	0.21*∗*^1^	0.28*∗∗*	0.34*∗∗*

Task completion	0.41*∗∗*	0.09	0.42*∗∗*	0.41*∗∗*

BRI	0.38*∗∗*	-0.02	0.28*∗∗*	0.33*∗∗*

MI	0.45*∗∗*	0.16	0.46*∗∗*	0.48*∗∗*

GEC (BRI+MI)	0.46*∗∗*	0.08	0.41*∗∗*	0.45*∗∗*

Behavioural shift	0.26*∗∗*	0.01	0.20*∗*	0.23*∗∗*

Cognitive shift	0.32*∗∗*	0.04	0.34*∗∗*	0.34*∗∗*

*∗*p<0.05, *∗∗*p<0.001

^1^ correlation significant in boys (r=0.3, p<0.01), but not in girls (r=0.1, p>0.05); other correlations mentioned in this table are statistically significant in both sexes.

## Data Availability

The data used to support the findings of this study are available from the corresponding author upon request.
